# Supplementary material for: Mycobacterium tuberculosis partitions the Krebs cycle under iron starvation
Source: eLife. 2026 Jun 25;14:RP107596. doi: 10.7554/eLife.107596 (PMC13299605; doi:10.7554/eLife.107596)
Supplement: Peer review history

# Editor report: eLife Assessment

**DOI:** 10.7554/eLife.107596.3.sa0

**Authors:** Bavesh D Kana

**Affiliations:** University of the Witwatersrand South Africa

**Keywords:** Solid, Valuable

This well-designed, **valuable** study uses isotope tracing to analyse how iron limitation alters TCA cycle metabolism in *Mycobacterium tuberculosis*, revealing potential antibiotic targets for non-replicating bacteria in the host. The evidence is **solid**, providing insights into metabolic remodelling under iron-limited conditions.

# Referee report: Reviewer #1 (Public review)

**DOI:** 10.7554/eLife.107596.3.sa1

*M. tuberculosis* exhibits metabolic flexibility, enabling it to adapt to various environmental stresses, including antibiotic treatment. In this manuscript, Serafini et al. investigate the metabolic remodeling of *M. tuberculosis* used to survive iron-limited conditions by employing LC-MS metabolomics and 13C isotope tracing experiments. The results demonstrate that metabolic activity in the oxidative branch of the TCA cycle slows down, while the reductive branch is reverted to facilitate the biosynthesis of malate, which is subsequently secreted.

Overall, this study is experimentally well-designed, particularly the use of 13C isotope tracing to monitor TCA cycle remodeling under iron-limited conditions. The findings are valuable as they offer potential new targets for antibiotics aimed at non-replicating *M. tuberculosis* occurring in the hosts.

Comments on revised version:

All concerns are well addressed.

I have one minor concern: Page 3 line 16 - Fig. 1G & H: The kinetics of ATP levels between H37Rv and Erdman seem different; Erdman induces greater ATP at days 2 and 3 after DFO treatment, which was not clear in H37Rv. Fig. 1I shows NAD/NADH ratio not NADH/NAD ratio. Please change it to NADH/NAD+ to be consistent with Supplement Fig. 1 result. Include the 17-day result of NADH/NAD+ in the discussion section to explain the different viability between the two strains.

# Referee report: Reviewer #2 (Public review)

**DOI:** 10.7554/eLife.107596.3.sa2

Summary:

The authors investigated the effect of prolonged iron limitation (which does stop growth but does not lead to cell death) alters central metabolism in *M. tuberculosis*. The major tool they used is metabolomics combined with stable isotope tracing. They show that the Krebs cycle is still active, despite the fact that it is dependent on some iron-dependent enzymes. They show that carbon flux through the oxidative branch of the Krebs cycle is stalled, resulting in the accumulation of metabolites, such as malate and alpha-ketoglutarate that are partially secreted. Apparently, the carbon flux from glycolysis is partially diverted to the reductive branch of the Krebs cycle. This is not achieved by using the glyoxylate shunt but probably through the GABA shunt. This unprecedented split of the Krebs cycle and malate secretion allows a continuous flow of carbon through the core of carbon metabolism, overcoming the metabolic stalling triggered by iron starvation.

Strengths:

Novel insight in the central metabolism of a major pathogen and its adaptation to iron starvation. Carefully conducted experimentation. Paper ends with a clear and helpful model.

Weaknesses:

The authors show some surprising and important findings, but would need a little more effort to really substantiate this. Especially the role of the GABA shunt should be genetically tested, as they did for ICL and the glyoxylate shunt.

Also, the dataset 1 is not very convincing, it is only based on transcriptomics and shown with up or down, hardly a strong base for major conclusions. The very least you want is actual differences, preferable on the protein level, where it really counts....

Comments on the revised version:

In the revised version all these points were appropriately dealt with and discussed, although some of them textually and not experimentally, but for reasons that are logical.

# Author comment: Author response

**DOI:** 10.7554/eLife.107596.3.sa3

**Authors:** Agnese Serafini, Acely Garza-Garcia, Davide Sorze, Luiz Pedro S de Carvalho, Riccardo Manganelli

**Affiliations:** University of Padua Padova Italy; Bind Research London United Kingdom; University of Padova Padova Italy; UF Scripps Institute for Biomedical Innovation and Technology Jupiter United States; University of Padova Padova Italy

The following is the authors’ response to the original reviews.

**eLife Assessment**

This well-designed, valuable study uses isotope tracing to analyse how iron limitation alters TCA cycle metabolism in *Mycobacterium tuberculosis*, revealing potential antibiotic targets for non-replicating bacteria in the host. The findings provide insights into metabolic remodelling under iron-limited conditions. Whilst some of the evidence is solid, the data around the GABA shunt is incomplete, requiring genetic validation, as was done for the glyoxylate shunt. Questions remain about the underlying mechanisms and their specific role in *M. tuberculosis* pathogenesis.

We thank the Editor and the reviewers for the positive evaluation of our work and for the constructive comments, which helped us improve the manuscript. We have carefully considered all the points raised and addressed them to the best of our ability. Regarding the GABA shunt, we acknowledge that genetic validation would significantly strengthen our conclusions; as this was not feasible within the revision timeframe, we have revised the relevant section by adopting more cautious language and have included genetic validation among the future perspectives. Additionally, we have expanded the discussion to address the relevance of our findings in the context of *Mtb* pathogenesis and host-pathogen interaction. A point-by-point response to each comment is provided below.

We also made minor adjustments to the main text and figures:

We removed “normalised” from the Y-axis of Figure 1 (the data are normalised and the procedure is described in the Materials and Methods).

We rearranged the order of a paragraph in the Introduction: the first paragraph “During infection pathogenic bacteria […] extensively investigated” has been moved down, (page 2, lines 8-12). -We edited two sentences in the Introduction (page 2, lines 4-7)

Supplementary Information: we added the following sentence at page 4, lines 23-24: “The probability of the Figure 3 and 4–figure supplement 1E scenario should be equivalent to that of the Figure 3 and 4–figure supplement 1F scenario.”

We made minor typing adjustments: page 3, lines 30 and 31; page 4, lines-11-12, lines 22-24; page 5, lines 23-24; page 7, line 6; page 12, lines 28 and 32.

We added details to the Materials and Methods section at page 17, lines 1 and 19-21.

**Public Reviews:**

**Reviewer #1 (Public review):**

*M. tuberculosis* exhibits metabolic flexibility, enabling it to adapt to various environmental stresses, including antibiotic treatment. In this manuscript, Serafini et al. investigate the metabolic remodeling of *M. tuberculosis* used to survive iron-limited conditions by employing LC-MS metabolomics and 13C isotope tracing experiments. The results demonstrate that metabolic activity in the oxidative branch of the TCA cycle slows down, while the reductive branch is reverted to facilitate the biosynthesis of malate, which is subsequently secreted.

Overall, this study is experimentally well-designed, particularly the use of 13C isotope tracing to monitor TCA cycle remodeling under iron-limited conditions. The findings are valuable as they offer potential new targets for antibiotics aimed at non-replicating *M. tuberculosis* occurring in the hosts. However, despite these strengths, the reviewer has concerns regarding the mechanistic basis underlying the observed metabolic remodeling and its role in *M. tuberculosis* pathogenesis.

We thank the reviewer for the positive evaluation of our work and for the constructive comments. Regarding the role of the observed metabolic remodelling in *Mtb* pathogenesis, we have expanded the discussion to address this aspect, contextualising our findings within the framework of *Mtb* infection and host-pathogen interaction (page 13, line 28-37; page 14, lines 1-23). Detailed responses to each specific comment are provided below.

Major comments

The authors argue that iron starvation is a physiologically relevant stressor encountered by *M. tuberculosis* post-infection. Using Erdman and H37Rv strains under DFO conditions, Erdman loses viability, whereas H37Rv maintains it. Nonetheless, both strains exhibit similar metabolic remodeling in the TCA cycle based upon metabolomics and isotope tracing data. The authors should clarify the specific metabolic adaptations in H37Rv that enable it to sustain viability under DFO conditions.

We thank the reviewer for this observation. Following additional experiments performed in response to subsequent comments, we re-analysed the secreted metabolite data and monitored ATP, NADH, and NAD^+^ levels over 17 days in both the Erdman and H37Rv strains. The results were concordant between the two strains, supporting the hypothesis that the decrease in CFU/mL over time does not reflect a loss of viability, but rather entry into a non-culturable state or, alternatively, an increased tendency to aggregate in liquid culture. Comments have been added at page 3, lines 16-24 and page 5, lines 30-36

A mechanistic explanation of how Mtb sustains viability under iron starvation is provided at page 13, lines 2837.

The authors report no significant changes in NAD/NADH and ATP levels in H37Rv and Erdman exposed to DFO conditions. They observe TCA cycle remodeling, particularly the reversal of the reaction between OAA and MAL, catalysed by malate dehydrogenase, an enzyme that uses NAD+ and NADH as cofactors. The directionality of this reaction likely depends on the relative levels of NAD+ and NADH. Additionally, other dehydrogenases, such as pyruvate DH and aKG DH, also require NAD+/NADH cofactors.

We thank the reviewer for this important observation. We agree that the directionality of the malate dehydrogenase reaction, as well as the activity of other NAD^+^/NADH-dependent dehydrogenases, is likely influenced by the redox state of the cell. We therefore measured the NADH/NAD^+^ ratio over 17 days in both strains under DFO conditions. We also note that the Y-axis title in Figure 1 was incorrectly reported and has been corrected accordingly. Results and interpretation of these new data are provided at:

page 3 lines 16-21

page 11 lines 16-36

page 12 lines 1-9

page 13 lines 3-5

In Figure 1I, NAD+ and NADH levels are monitored only at day 3 post-exposure to DFO conditions. Since Erdman loses viability after 2-3 weeks, the authors should include measurements of NAD+, NADH, and ATP levels at weekly intervals up to 3 weeks.

We thank the reviewer for this suggestion. As recommended, we extended the monitoring of NAD^+^, NADH, and ATP levels over 17 days in both strains. Results and interpretation have been discussed together and are reported in the manuscript. Please refer to the response above for the relevant page and line references.

Furthermore, glycine levels - which are linked to NAD+ recycling via the conversion of glyoxylate - should be measured under both HI and DFO conditions as an indirect indicator of the NAD+/NADH ratio.

We thank the reviewer for this comment. However, we believe that glycine levels cannot be considered a reliable indirect indicator of the NAD^+^/NADH ratio, as glycine is involved in multiple metabolic pathways. It can originate from serine, threonine, glyoxylate, or protein degradation, and can be incorporated into proteins, degraded to CO_2_ and NH_4_^+^, converted to glyoxylate, or transformed into other amino acids. Due to its metabolic versatility, therefore, glycine levels lack the specificity required to reliably reflect the cellular NAD^+^/NADH ratio. In addition, we could not find a single study that claim that glycine levels can be used as indicators of NAD^+^/NADH ratio.

Nevertheless, this comment prompted us to examine glycine levels and isotopologue distribution under iron deprivation. Glycine levels showed no consistent trend under DFO conditions, remaining unchanged or increasing in both the Erdman and H37Rv backgrounds.

Importantly, the isotopologue distribution analysis led us to conclude that glyoxylate is not a key precursor of glycine under iron starvation. This new analysis is described at page 10 (lines 1-20), and a new supplementary figure has been added, Figure 3 and 4 – figure supplement 3.

In Figure 2A, it is unclear why a 100-fold accumulation of aKG does not correspond proportionally to the accumulation of (iso)citrate.

We thank the reviewer for this observation. We agree that this point required clarification and have added a comment addressing this apparent discrepancy in the main text at page 4, lines 12–17.

The authors state that fumarate, aKG, (iso)citrate, malate, and pyruvate are secreted under DFO conditions. While the secretion of aKG and pyruvate makes sense, given their marked intracellular accumulation, it is puzzling why (iso)citrate, malate, and fumarate are secreted even though there are no changes in their intracellular abundance.

To rule out the possibility that these metabolites are released due to bacterial lysis rather than active secretion, the authors should analyze the 13C-labeled fractions of these metabolites in the culture filtrate using the *M. tuberculosis* culture in media containing 13C glycerol.

We thank the reviewer for this important observation.

Regarding the possibility of cell lysis, although it cannot be completely ruled out, several observations indicate that the increase in extracellular malate was not due to lysis. If substantial cell lysis had occurred, we would expect a general increase in all extracellular metabolites. However, the extracellular fumarate and succinate levels remained unchanged in both strains under DFO (similarly to the control conditions, HI and LI). Glutamate was detected in the culture filtrate, but its abundance increased only under HI conditions, not under DFO, in either H37Rv or Erdman. The lack of increase in extracellular glutamate, fumarate and succinate, therefore suggests that, even if some cell lysis occurred, it was minimal and did not significantly affect our observations.

Regarding the 13C-fractions, we note that it is unclear how should the labelling profile would differ if extracellular metabolite derived from cell lysis. Nevertheless, as suggested by the reviewer, we compared the labelled fractions of extracellular isocitrate, malate, fumarate and glutamate. The comparison revealed variations consistent with two blocks in the carbon flow occurring at the levels of pyruvate and alpha-ketoglutarate, resulting in a slowdown in the downstream flux.

A description of these new considerations has been added at page 5 (lines 27-36) including the Figure 2 – figure supplement 2 and a new section of SI-Appendix. Therefore, we are confident that the selective appearance of some but not all metabolites in culture filtrates is consistent with secretion but not cell lysis.

To validate the role of the PCK-mediated reductive TCA cycle in malate biosynthesis and secretion under DFO conditions, the authors should generate a malate dehydrogenase (MDH) knockdown strain, considering that MDH is essential, and examine the 13C labeling patterns and NAD/NADH under DFO conditions.

The authors also observe decreased GABA abundance and overall 13C labeling in DFO conditions, suggesting that the GABA shunt is the primary route for succinate biosynthesis under DFO conditions. Thus, it is strongly recommended that the authors perform a 13C glutamate tracing experiment to directly track labeling in aKG and GABA shunt metabolites, providing more definitive evidence for the involvement of the GABA shunt.

We thank the reviewer for these valuable suggestions. We fully agree that both experiments would significantly strengthen the conclusions of our work.

Regarding the MDH knockdown strain, we acknowledge that this experiment would provide direct validation of the PCK/PCA-mediated reductive TCA cycle in malate biosynthesis. However, generating a knockdown strain in Mtb is a technically demanding and time-consuming process, requiring several months even under optimal conditions, which makes it unfeasible within the revision timeframe. We have therefore incorporated this experiment as a future perspective in the conclusions, highlighting its importance for further validating the proposed model.

Regarding the GABA shunt, we took the reviewer's comment as an opportunity to critically re-evaluate the strength of our data. As a result, we have revised the manuscript by merging the GABA shunt discussion with the glyoxylate shunt section, while adopting more cautious language in the concluding statement to reflect its hypothetical nature. The related figures have been moved to the Supplementary Materials. These aspects have been included among the future perspectives in the conclusions. Page 11, lines 10-13; page 14, lines 3-7.

**Reviewer #2 (Public review):**

Summary:

The authors investigated the effect of prolonged iron limitation (which does stop growth but does not lead to cell death), altering central metabolism in *M. tuberculosis*. The major tool they used is metabolomics combined with stable isotope tracing. They show that the Krebs cycle is still active, despite the fact that it is dependent on some iron-dependent enzymes. They show that carbon flux through the oxidative branch of the Krebs cycle is stalled, resulting in the accumulation of metabolites, such as malate and alphaketoglutarate, that are partially secreted. Apparently, the carbon flux from glycolysis is partially diverted to the reductive branch of the Krebs cycle. This is not achieved by using the glyoxylate shunt but probably through the GABA shunt. This unprecedented split of the Krebs cycle and malate secretion allows a continuous flow of carbon through the core of carbon metabolism, overcoming the metabolic stalling triggered by iron starvation.

Strengths:

Novel insight into the central metabolism of a major pathogen and its adaptation to iron starvation. Carefully conducted experimentation. The paper ends with a clear and helpful model.

Weaknesses:

The authors show some surprising and important findings, but they would need a little more effort to really substantiate these. Especially the role of the GABA shunt should be genetically tested, as they did for ICL and the glyoxylate shunt.

We thank the reviewer for the positive evaluation of our work. We agree that genetic validation of the GABA shunt would significantly strengthen our conclusions. However, generating the required mutant strains in Mtb is a technically demanding and time-consuming process that is unfeasible within the revision timeframe. In light of this, we have revised the manuscript by merging the GABA shunt discussion with the glyoxylate shunt section. This reorganization contextualizes the GABA shunt within a broader discussion, while adopting more cautious language in the concluding statement to reflect its hypothetical nature. Future genetic validation, including the generation of appropriate mutant strains, has been included among the future perspectives in the conclusions.

Page 11, lines 10-13; page 14, lines 3-7.

Also, dataset 1 is not very convincing, it is only based on transcriptomics and shown with up or down; this is not a strong base for major conclusions. As a minimum, one would want actual differences, preferably on the protein level, where it really counts.

We thank the reviewer for this comment. We would like to clarify that Dataset S1 compiles transcriptomic and proteomic data from previously published studies, which represent the rational basis of our investigation. These data are consistently cited throughout the manuscript. The dataset was included solely as a convenience tool for the reader, to provide easy access to the relevant published information. To avoid any misunderstanding regarding its scope, we have renamed the file to 'Dataset S1 - Publicly available transcriptomic datasets referenced in this study'. Our conclusions derive from the integration of these published data with the novel biochemical and metabolomic evidence generated in this study. Further, to assist the reading, we added a clarifying description at top of “DE” column.

**Recommendations for the authors:**

**Reviewer #1 (Recommendations for the authors):**

(1) Clarify the definitions of "growth defect" and "growth arrest" under LI and DFO conditions, respectively.

(2) In Figure 2A, specify the unit of the y-axis. Is it on a log scale?

(3) Raw data of metabolomics and 13C isotope tracing experiments should be either deposited in public websites or provided as a separate file.

We thank the reviewer for these comments.

Regarding the definition of 'growth defect' and 'growth arrest': we replaced 'defect' with 'slowdown' to better reflect the observed phenotype under LI conditions.

Regarding Figure 2A: we have specified the unit of the Y-axis and clarified whether the scale is logarithmic in the figure legend. We have done that for all the figures containing charts with Y/X axis in logarithmic scale. We added secondary tick marks in the charts of Figure 5G.

Regarding raw data availability: the metabolomics data have been deposited in the Zenodo database. The reference number has been added to the manuscript."

**Reviewer #2 (Recommendations for the authors):**

It is mentioned that measurement of the activity of these two enzymes in cell-free extracts revealed the presence of PCA activity in the DFO condition (Figure 5E), but not of MEZ activity (data not shown). Activity measurements are a great added value, but then activities should be shown, also for MEZ.

We thank the reviewer for this suggestion. We agree that showing enzyme activity data adds value to the manuscript. As recommended, activity measurements have been included in the supplementary materials (Figure 5 – figure supplement 1).
